# Scalable Large-Area 2D-MoS_2_/Silicon-Nanowire Heterostructures for Enhancing Energy Storage Applications

**DOI:** 10.1021/acsaem.3c03055

**Published:** 2024-03-07

**Authors:** Ioannis Zeimpekis, Tasmiat Rahman, Oi Man Leung, Jack Tyson, Martin Ebert, Stuart A. Boden, Carlos Ponce De Leon, Katrina A. Morgan

**Affiliations:** † Electronics and Computer Science, University of Southampton, Southampton SO171BJ, U.K.; ‡ Faculty of Engineering and Physical Sciences, Mechanical Engineering department, University of Southampton, Southampton SO171BJ, U.K.

**Keywords:** 2D materials, energy storage, MoS_2_, nanowires, scalable, TMD

## Abstract

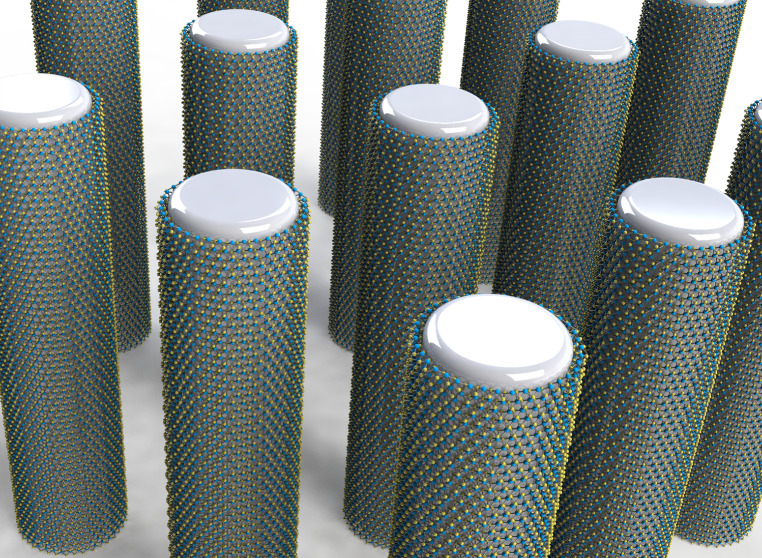

Two-dimensional (2D) transition-metal dichalcogenides have shown great potential for energy storage applications owing to their interlayer spacing, large surface area-to-volume ratio, superior electrical properties, and chemical compatibility. Further, increasing the surface area of such materials can lead to enhanced electrical, chemical, and optical response for energy storage and generation applications. Vertical silicon nanowires (SiNWs), also known as black-Si, are an ideal substrate for 2D material growth to produce high surface-area heterostructures, owing to their ultrahigh aspect ratio. Achieving this using an industrially scalable method paves the way for next-generation energy storage devices, enabling them to enter commercialization. This work demonstrates large surface area, commercially scalable, hybrid MoS_2_/SiNW heterostructures, as confirmed by Raman spectroscopy, with high tunability of the MoS_2_ layers down to the monolayer scale and conformal MoS_2_ growth, parallel to the silicon nanowires, as verified by transmission electron microscopy (TEM). This has been achieved using a two-step atomic layer deposition (ALD) process, allowing MoS_2_ to be grown directly onto the silicon nanowires without any damage to the substrate. The ALD cycle number accurately defines the layer number from monolayer to bulk. Introducing an ALD alumina (Al_2_O_3_) interface at the MoS_2_/SiNW boundary results in enhanced MoS_2_ quality and uniformity, demonstrated by an order of magnitude reduction in the B/A exciton photoluminescence (PL) intensity ratio to 0.3 and a reduction of the corresponding layer number. This high-quality layered growth on alumina can be utilized in applications such as for interfacial layers in high-capacity batteries or for photocathodes for water splitting. The alumina-free 100 ALD cycle heterostructures demonstrated no diminishing quality effects, lending themselves well to applications that require direct electrical contact with silicon and benefit from more layers, such as electrodes for high-capacity ion batteries.

## Introduction

There is currently a rapidly growing demand for low-cost, high-density energy storage.^1^ This is especially prevalent as the world moves toward clean energy that requires storage to be effectively deployed. This must be performed in a sustainable manner; therefore, alternative electrodes and chemistries are currently being rapidly explored. The unique chalcogen–metal–chalcogen structure of transitional-metal dichalcogenides (TMDs) gives them unique properties such as the ability to retain large numbers of ions due to their interlayer spacing^2,3^ (2H-MoS_2_ 6.5 Å,^4^ 1T-MoS_2_ > 10 Å,^5^ tunable through addition of elements^6,7^) and their intralayer stackable nature. TMDs can facilitate increased ionic interaction and increase electrical conduction within electrode/electrolyte interfaces in ionic batteries, leading to high-capacity batteries.^4,7,8^ The ability to provide high capacity coupled with the high stability of these materials constitutes them as ideal candidates for battery electrodes and interfacial layers.^9−11^ Other TMD properties including high mobility, tunable bandgap, high mechanical flexibility, high surface-to-volume ratio, and supercapacitive and catalytic activity offer unique property combinations for next-generation fuel cells^12,13^ and solar cells.^13,14^ Specifically, TMDs can act as power conversion efficiency enhancing layers in solar cells,^15,16^ additionally increasing lifetime and dramatically reducing waste arising from solar panel replacement. TMDs can also be used in the cost-effective production of hydrogen,^11,17,18^ resolving one of the most persistent stumbling blocks for its use as a clean energy source.

The energy storage and generation potential of TMDs can be further exploited by increasing their acting surface area. This inherently enhances electrolyte/semiconductor interactions, electrolyte/electrode interactions, charge carrier transport efficiency, light-trapping phenomena, and scattering effects and thus increases performance at the device level.^19−22^ High-surface-area 2D material structures have been successfully demonstrated using silicon nanowires and similar morphologies, as a scaffold.^19−22^ However, due to the fabrication complexity of large-area 2D materials across high-aspect-ratio structures, accurate, commercially scalable, parallel layer growth of 2D-MoS_2_ layers on silicon nanowires has not been demonstrated to date.

2D transition-metal dichalcogenides, such as MoS_2_, can be produced using a variety of deposition and growth techniques including sputtering,^23,24^ thermal treatments,^25,26^ drop-casting,^21,27^ inkjet printing,^28,29^ chemical vapor deposition (CVD),^30−32^ and ALD^33,34^ or a combination of these. The MoS_2_ layer number defines the electrical properties such as conductance and the ability to make good contacts determining the performance and application space. Direct sputtering of MoS_2_ on silicon nanowires results in amorphous growth, unwanted material build-up on the tips of the wires, and vertical layer growth to the nanowire surface,^20^ limiting performance. Drop-casting faces similar challenges, including inaccurate control of the number of layers.^21^ CVD offers a more controllable and scalable method, producing conformal growth with parallel layers,^35^ but the exposure of silicon nanowires to a harsh sulfur environment reduces the quality of the system.

To investigate the performance of high aspect ratio MoS_2_-coated electrodes, we employ a scalable method to fabricate large areas of high aspect ratio silicon nanowires^36−38^ (height 3.5 μm, width 100 nm) conformally coated with MoS_2_ in parallel growth orientation, with no nanowire deterioration. MoO_3_ is deposited by ALD to create a template and then converted to MoS_2_ in a sulfur environment annealing.^39^ The template layer protects the substrate, resulting in a higher-quality hybrid structure. This ALD-based method provides excellent conformity, uniformity, crystallinity, and decoupled stoichiometric and layer number control. Following our previous work, we investigate the addition of an interfacial alumina layer to further enhance the quality of subsequent layers.^40^ Our large-area industrially scalable and highly controllable hybrid structure approach aims among other applications to provide high-performance electrodes for batteries^41,42^ to underpin high capacity, long lifetime, and high safety. Our results include scanning electron microscopy (SEM), TEM, Raman spectroscopy, and photoluminescence to analyze the MoS_2_ layer number, quality, uniformity, and interfacial effects of the alumina.

## Experimental Section

To investigate the individual fabrication process step effects on the nanowire substrates and to gain understanding of the significance of the process order, we fabricated seven samples as shown in Table 1. These 2.5 × 2.5 cm SiNW samples were fabricated as described, undergoing various steps of the MoS_2_ growth using our two-step process. One sample (S1) was used as a SiNW reference and underwent no further processing. Two samples underwent one of the two steps of the MoS_2_ growth, separately, with one sample (S2) undergoing the hydrogen sulfide (H_2_S) anneal with no prior MoO_3_ growth, while the other (S3) underwent the standard 15 ALD cycles of MoO_3_ growth and no anneal. Please note, throughout the manuscript, 15 and 100 cycles refer to the number of ALD cycles used to deposit the MoO_3_; these numbers do not refer to the number of subsequent MoS_2_ layers that have grown as a result of the process. Please see the Raman results for layer number. Two further samples underwent the full MoS_2_ process, one with 15 ALD cycles of MoO_3_ (S4) and one with 100 ALD cycles of MoO_3_ (S5), both followed by the H_2_S anneal. The last two samples (S6 and S7) underwent the same process but with an interim ALD alumina step prior to the MoO_3_ growth.

**Table 1:** Sample List with Process Steps and Aims for Each Sample

Sample name	Process steps (post SiNW growth)	Investigations
S1	No further processing	Bare SiNW for reference
S2	Anneal	H_2_S anneal effects on SiNWs
S3	15 cycles of MoO_3_ ALD, no anneal	MoO_3_ ALD growth on SiNWs
S4	15 cycles of MoO_3_ ALD + H_2_S anneal	Monolayer MoS_2_ growth on SiNWs
S5	100 cycles of MoO_3_ ALD + H_2_S anneal	Multilayer MoS_2_ growth on SiNWs
S6	Al_2_O_3_ ALD + 15 cycles of MoO_3_ ALD + H_2_S anneal	Al_2_O_3_ interlayer for monolayer MoS_2_ on SiNWs
S7	Al_2_O_3_ ALD + 100 cycles of MoO_3_ ALD + H_2_S anneal	Al_2_O_3_ interlayer for multilayer MoS_2_ on SiNWs

### Silicon Nanowire Fabrication

The fabrication process starts from cleaving an n-type silicon wafer into 2.5 cm × 2.5 cm chips. These are then cleaned using RCA1 (H_2_O_2_–NH_4_OH–H_2_O) and RCA2 (H_2_O_2_–HCl–H_2_O) solution, as well as a Piranha Etch (H_2_SO_4_–H_2_O_2_). Following the cleaning steps, they undergo a metal-assisted chemical etch (MACE) process to form the silicon nanowires. This is a top-down process, which begins with nucleation at the surface using Ag nanoparticles, followed by etching with hydrofluoric acid.^37,38^ This uses a solution of AgNO_3_ and hydrofluoric (HF) acid and leaves grass-like nanowires, whereby the height is determined from the etch time and density from the AgNO_3_ concentration. Post etching, the remaining Ag nanoparticles are removed using nitric acid. These silicon surfaces have ultralow broadband reflectivity (<1%) due to their high surface area (therefore referred to as black-Si) and have been utilized for energy harvesting, storage, and sensing.

### Alumina Interfacial Deposition

Alumina is deposited via ALD using a Veeco Savannah S200 with TMA and water vapor as the precursors at 200 °C.

### Two-Step MoS_2_ Growth

MoS_2_ is grown in a two-step process; the first step is an ALD MoO_3_ layer, and the second step is an anneal in hydrogen sulfide (H_2_S) whereby the MoO_3_ is converted to MoS_2_ as described in ref (39). We employed two different thicknesses of MoO_3_ at the ALD stage, thus leading to two different MoS_2_ layer thicknesses. This was attained by controlling the number of ALD cycles to either 15 or 100. The cycle number refers to the ALD growth mechanism, whereby one cycle involves the input and purge of two gases, leading to a self-limiting growth technique. This accurate control of the layer thickness enables uniform growth and high-quality layers.

### Characterization Techniques

The characterization techniques used to assess the MoS_2_ growth on silicon nanowires in this work are Raman, photoluminescence, and TEM, enabling the presence, quality, coverage, and layer number of MoS_2_ to be confirmed, and SEM, allowing the structural stability to be investigated.

SEM was performed using a Jeol JSM 7500F field emission scanning electron microscope (FESEM) and a Zeiss Nvision40 SEM, with accelerating voltage between 2 and 5 kV. Raman and PL were performed using a Renishaw InVia microscope, with a 532 nm laser, for 10 s irradiation time, with a laser power of 0.55 mW at the sample (1% of 55 mW on sample using a 100 mW laser), at a ×50 objective, for three accumulations per measurement.

Scanning TEM (STEM) micrographs were taken with a Thermo Fisher Scientific (formerly FEI) TEM Titan3 80–300 microscope at 300 kV acceleration voltage. The microscope is equipped with a cs-aberration corrector for the TEM imaging mode. Image acquisition in TEM mode was done with a CCD camera (2k × 2k). STEM micrographs were acquired by using a high-angle annular dark-field (HAADF) detector. Samples were glued into the resin and mechanically polished, followed by ion beam milling for thinning.

## Results and Discussion

### SEM

**Figure 1:**
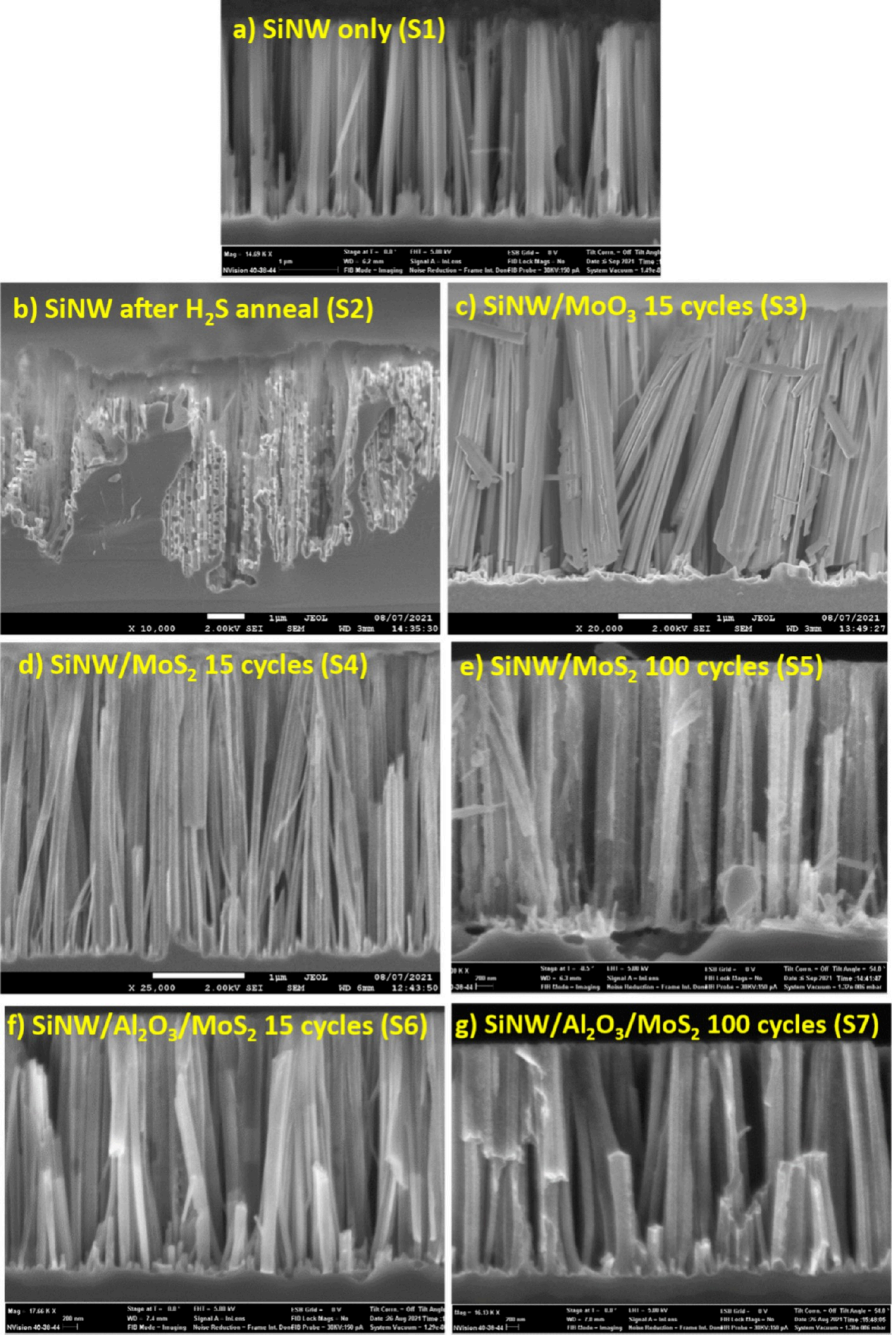
SEM cross-sectional images of SiNWs at different 2D MoS_2_ process steps, corresponding to samples from Table 1: (a) reference sample of SiNW with no further processing (S1), (b) SiNW exposed to H_2_S anneal with no MoO_3_ layer (S2), (c) SiNW with MoO_3_ via ALD but no anneal (S3), (d) 15 cycles of MoO_3_ ALD followed by H_2_S anneal (S4), (e) 100 cycles of MoO_3_ followed by H_2_S anneal (S5), (f) alumina interface with 15 cycles of MoO_3_ ALD followed by H_2_S anneal (S6), and (g) alumina interface with 100 cycles of MoO_3_ followed by H_2_S anneal (S7).

Figure 1 shows the SEM results of the nanowires at varying stages of the fabrication process and for the different splits in the batch, i.e., those with and without alumina, and for two different MoS_2_ thicknesses, as determined by the ALD cycle number: 15 and 100. The SEM image (Figure 1(a)) shows the reference sample of SiNWs, with no processing (S1). Figure 1(b) shows the destructive result on silicon nanowires that occurs after the sample is annealed in H_2_S when the protective MoO_3_ layer is not applied (S2). This demonstrates that H_2_S aggressively reacts with Si when exposed directly. It is for this reason that direct growth of MoS_2_ onto silicon is incredibly challenging, especially when using traditional CVD processes which directly expose the substrates to H_2_S. To overcome this, our two-step MoS_2_ process deposits MoO_3_ first (Figure 1(c)) (S3), followed by a H_2_S anneal. The deposition of the MoO_3_ layer first acts as a protection layer to the substrate below, thus facilitating direct growth of MoS_2_ onto silicon, with no apparent damage to the nanowire structure below, as shown in Figure 1(d) (S4). This validates the compatibility of using this ALD growth process of MoS_2_ with nanowires, based on the nonevident negative effects.

Figure 1(d–g) demonstrates samples S4–S7, respectively, with and without alumina and for the thick and thin MoS_2_ layers. While there are no discernible differences between these samples due to the resolution of the SEM, these images still show no structural damage has occurred at any point of our process flow, verifying the fabrication steps are compatible with the SiNWs.

### Raman

**Figure 2:**
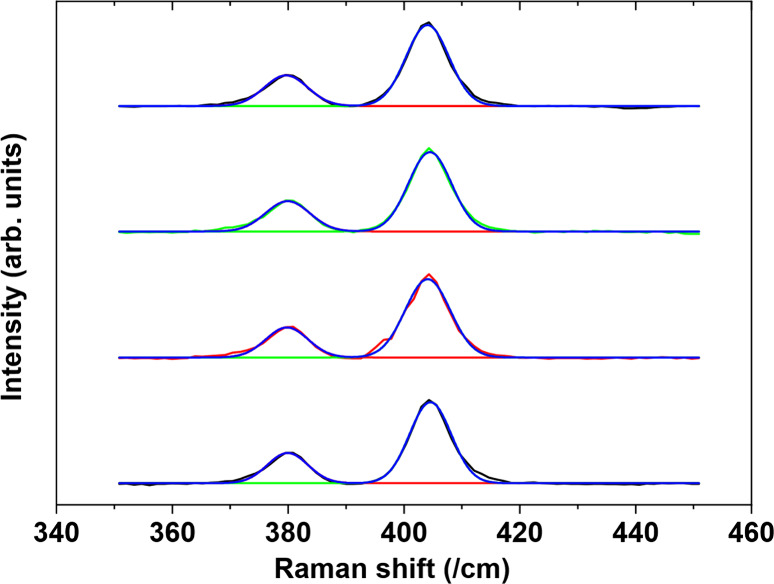
Typical Raman spectra of four points taken on S4 (15 ALD cycles of SiNW/MoS_2_), highlighting the MoS_2_ lower peak of E_2g_ and the higher peak of A_1g_.

To confirm successful growth, ascertain the presence of MoS_2_ on the silicon nanowires, and measure the number of layers, Raman spectroscopy was performed. Two characteristic MoS_2_ peaks, the E_2g_ and A_1g_ at a Raman shift of 380 and 405 cm^–1^, were seen on all MoS_2_ samples (S4 to S7), demonstrating successful growth of MoS_2_ on silicon nanowires. Figure 2 is a representative spectrum of all samples with four Raman measurements taken across four spatial locations across the samples. This provided each sample with an average and standard deviation, using a Gaussian fitting function for the peaks.

**Figure 3:**
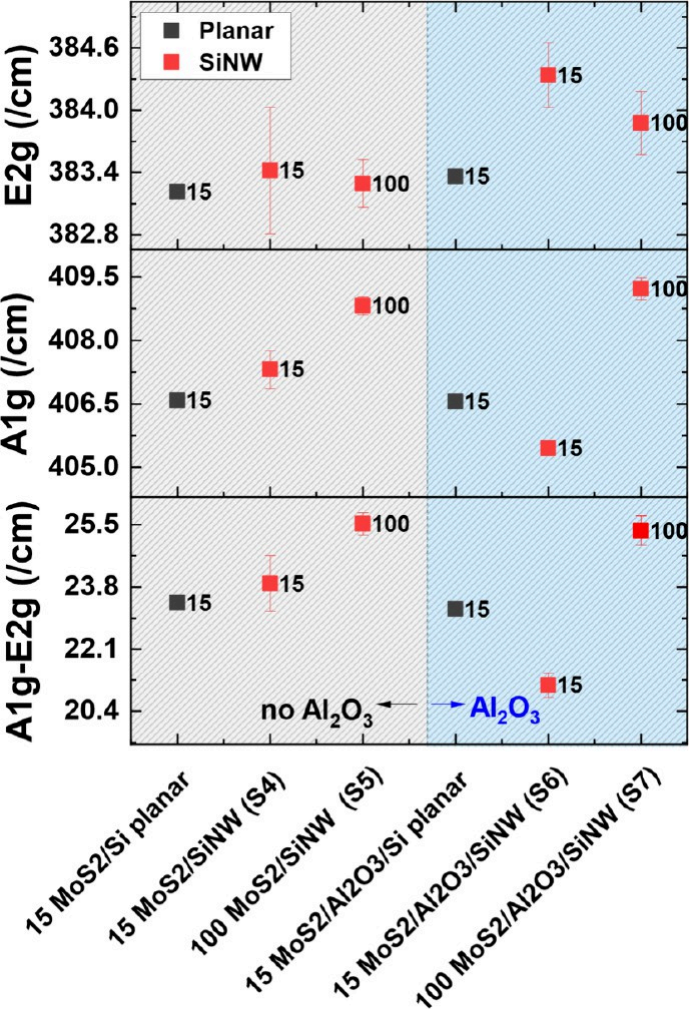
Raman E_2g_ and A_1g_ peak locations, with peak difference shown for SiNW-MoS_2_ stacks, with and without alumina for 15 and 100 ALD cycles. A planar reference sample is included.

The Raman peak locations and peak separation of four MoS_2_ samples, two with and two without Al_2_O_3_, with thick and thin layers (S4 to S7) (15 and 100 ALD cycles of MoO_3_, converted to MoS_2_) are shown in Figure 3. For both samples with 100 cycles of ALD (S5 and S7), a significant increase in peak separation is seen compared with their 15 cycle counterparts (S4 and S6). This increase in peak separation is expected for thicker samples, seen throughout the literature, and can be used to determine the number of layers.^43^

A peak separation of ∼25 cm^–1^ has been reported in the literature to reflect five to six layers^43−45^ of MoS_2_, with some literature reporting this tends toward “bulk” material behavior.^46−48^ This is what we see for the thicker 100 ALD cycle samples in this work.

The 15 cycle ALD samples (S4 and S6) demonstrate a peak separation of ∼24 cm^–1^, reported to be four MoS_2_ layers or below, with SiNW/Al_2_O_3_/MoS_2_ (S6) exhibiting the smallest separation at ∼20 cm^–1^, indicating one to two layers of MoS_2_.^47,49^ The presence of Al_2_O_3_ in combination with the silicon nanowires appears to have resulted in the lowest number of MoS_2_ layers. A reason for this could be due to the alumina layer encapsulating the SiNW, acting as a moisture barrier and reducing water and oxygen molecules on the surface of the nanowires. This removal of additional molecules and creating this hydrophilic surface on the SiNWs could therefore remove an interfacial MoO_3_ layer that forms when the molybdenum precursor first enters the chamber on the first cycle. Where alumina is present and fully reacted, this hydrophilic-terminated surface would mean the first precursor injection does not react with any leftover oxygen and in turn will have a reduced number of subsequent MoO_3_ layers.^50^

The presence of MoS_2_ Raman peaks occurring across the spatial area of the sample indicates an ability to grow a single layer of MoS_2_ conformally on a structured sample across a large area. This, which can be scaled up to 300 mm wafer scales as the standard, is used in semiconductor fabrication plants across the world.^51,52^ Furthermore, the ability to directly grow MoS_2_ onto bare silicon (15 cycle SiNW/MoS_2_), as opposed to growing on silicon dioxide followed by a transfer process, will enable active electronic devices to be processed much more readily and with fewer defects due to the monolithic integration. While Raman has verified a good spatial uniformity at a chip level, TEM was also used to investigate the number of layers for the 15 cycle samples with higher accuracy and determine spatial uniformity across the nanowires and growth orientation.

### Transmission Electron Microscopy

**Figure 4:**
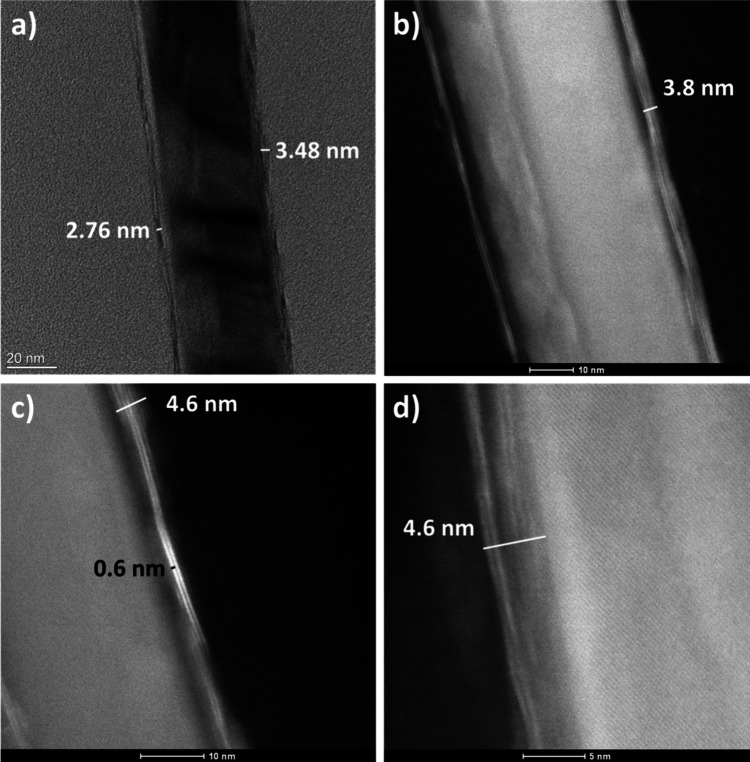
TEM images of nanowire cross sections from a 15 ALD cycles MoS_2_/SiNW (S4) sample with no alumina at varying magnifications: (a) ×115k TEM, (b) ×910k scanning TEM, (c) ×1300k scanning TEM, and (d) ×2.55 M scanning TEM. Individual layers of MoS_2_ can be seen on the nanowire surface, varying from two layers up to five and above.

TEM was performed on the samples with 15 ALD cycles only (S4 and S6) as these samples will display independent layers, which can be used to confirm Raman results. Figure 4 shows TEM cross sections of a nanowire for the 15 cycle MoS_2_/SiNW sample without alumina (S4). The images clearly show continuous parallel MoS_2_ coverage over the entire nanowire with between two and five layers. This indicates that there is successful growth along the full surface of the nanowire, matching well with the Raman measurement.

**Figure 5:**
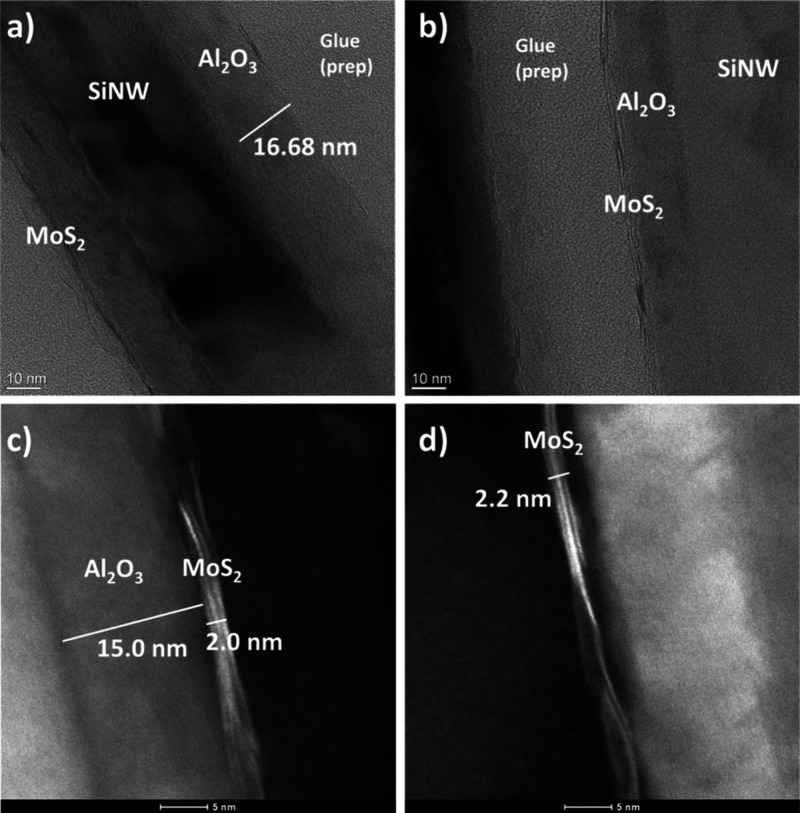
TEM images of nanowire cross sections from a 15 ALD cycles MoS_2_/Al_2_O_3_/SiNW (S) sample with alumina at varying magnifications: (a) ×45k TEM, (b) ×145k TEM, (c) ×1.8 M scanning TEM, and (d) ×1.8 M scanning TEM. A uniform alumina layer is visible with two layers of MoS_2_ distributed uniformly across the nanowire surface.

Figure 5 demonstrates TEM images of cross section nanowires from 15 ALD cycles of MoS_2_ on SiNWs with alumina (S6). The alumina is clearly present and uniform across the surface of the nanowire, averaging around 15 nm, as expected for this ALD recipe. The MoS_2_ layers are clearly present in these images, demonstrating two layers uniformly along the surface of the nanowires. This verifies the Raman results which indicated one to two layers. These TEM images indicate the uniformity and layer number are a lot more consistent across the surface of the nanowires compared to the sample without alumina present, in agreement with Raman results. This indicates that alumina acts as a passivating and seeding layer for higher-quality subsequent MoS_2_ growth.

### Photoluminescence

**Figure 6:**
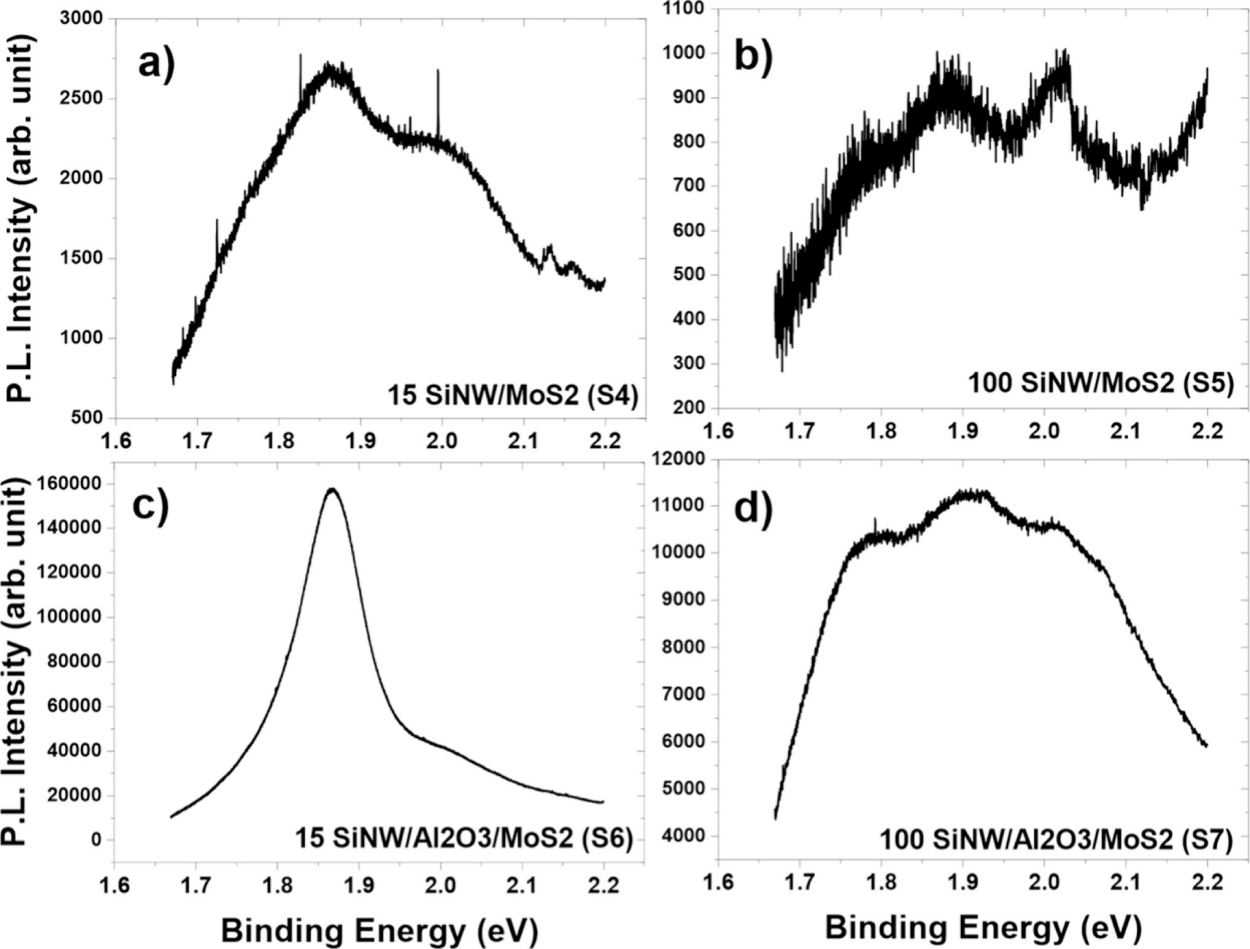
Raw photoluminescence data for SiNW-MoS_2_ samples, with measured intensity, for (a) 15 cycles of MoS_2_ without alumina (S4), (b) 100 cycles of MoS_2_ without alumina (S5), (c) 15 cycles of MoS_2_ with alumina (S6), and (d) 100 cycles of MoS_2_ with alumina (S7).

**Figure 7:**
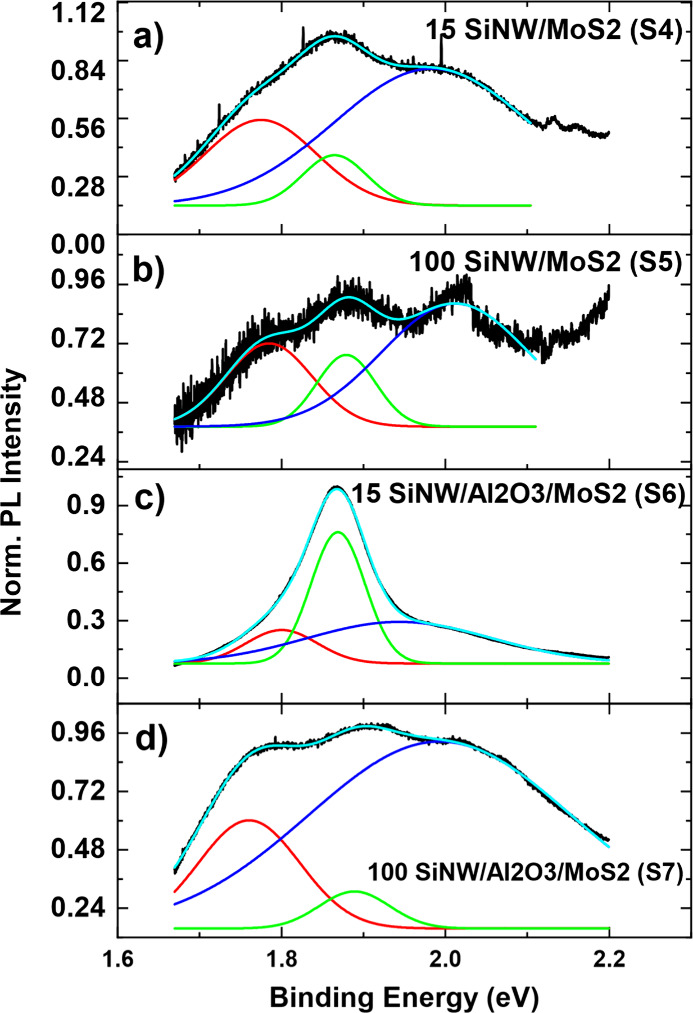
Normalized and fitted photoluminescence peaks of SiNW-MoS_2_ stacks for (a) 15 cycles of MoS_2_ without alumina (S4), (b) 100 cycles of MoS_2_ without alumina (S5), (c) 15 cycles of MoS_2_ with alumina (S6), and (d) 100 cycles of MoS_2_ with alumina (S7).

To confirm the presence of monolayers and ascertain the layer film quality, photoluminescence measurements were performed. The raw data of the four SiNW/MoS_2_ samples can be seen in Figure 6, whereby the photoluminescence intensity is plotted. The normalized PL plot for the samples is shown in Figure 7, normalized by the maximum intensity, and fitted with three Gaussian peaks that correspond to the peaks seen in typical MoS_2_ PL spectra.^53^ The green middle peak is the A exciton; the blue highest energy peak is the B exciton; while the red lowest-energy peak is identified as the A-Trion, often related to the presence of defects, doping, or substrate effects causing excess electron charges.^53^

The 15 cycle SiNW/Al_2_O_3_/MoS_2_ sample (S6) exhibits typical monolayer MoS_2_ PL spectra often seen in the literature, with a dominant A exciton peak at 1.87 eV and a small A-Trion and B exciton peak at 1.84 and 1.96 eV, respectively, and a B/A intensity ratio of 0.3.^54−56^ The higher absolute intensity of this sample compared to the others, as seen in Figure 6, might also indicate the presence of a stronger PL, indicating fewer MoS_2_ layers are present in comparison to the other samples as also shown through the TEM. The 15 cycle SiNW/MoS_2_ (S4) sample also exhibits the MoS_2_ peak shape as seen in the literature, although the high B/A peak intensity of nearly 3 indicates a higher defect density and thus lower MoS_2_ quality.^57^ This would suggest that the alumina layer promotes a better quality subsequent MoS_2_ film.

The 100 cycle samples (S5 and S7) exhibit a lower absolute intensity, which could be attributed to bulk-type behavior where an indirect band gap is present, rather than a direct one as for monolayer samples, thus losing the emergence of strong photoluminescence. Nonetheless, the B/A ratio is better for the 100-cycle sample without alumina (S5), at 1.7, than for the sample with alumina (S7), at 5.0, which indicates a reduction in MoS_2_ quality conferred by the alumina buffer for the thicker MoS_2_. This could be due to the alumina creating a better interfacial layer for the subsequent MoO_3_ growth, which is more prominent when the layers are thin but is dwarfed in thicker samples. In addition, the alumina layer could act as a protective layer for the silicon against the H_2_S anneal for the thin MoO_3_ samples, but this effect is unnecessary when the thicker MoO_3_ layer acts as a protective layer itself.

The trion peak is present in all four samples, indicating a typical excess of electrons as commonly reported in the literature for MoS_2_.^55,58,59^ The absolute intensity of the A-Trion peak is largest for the 100 cycles with alumina (S7) but is then followed by the 15 cycles without alumina (S4), with the smallest intensity coming from the 15 cycles with alumina (S6). Therefore, there does not appear to be a clear trend with Trion peak intensity and the substrate and cycle number.

The reduction in the A exciton intensity, along with the red-shifted and larger intensity A-Trion peak, has been reported in the literature for samples that are n-type.^60,61^ These features can be seen for both the 100 cycle samples (S5 and S7) and the 15 cycles without alumina (S4), when compared with the 15 cycles with alumina (S6).

We can conclude that the 15 cycles of ALD MoS_2_ with alumina (S6), with the highest PL A exciton peak intensity and lowest B/A peak intensity ratio, along with a nonredshifted and lower intensity A-Trion peak, demonstrate the sample with the least number of excess electrons and unintentional n-type doping and thus have the highest-quality MoS_2_. For the thickest samples, the opposite trend with alumina is seen. Therefore, the use of an alumina layer can be selected depending on the ideal thickness required for the specific application and whether an insulating layer is plausible. For example, if used as an electrode in an ion battery application, it may be better to have many layers so that a higher number of ions can be inserted, thus increasing battery capacity. As such, our results would indicate an alumina layer is not necessary for higher-layer numbers, as a better quality MoS_2_ is seen for thicker films directly grown onto SiNWs, which will also allow for the silicon to contribute higher electrical conductivity, potentially further enhancing battery performance. However, if MoS_2_ is to be used as a standalone layer that is to be electrically isolated from the substrate, a better quality fewer-layer MoS_2_ film could be achieved with an underlining alumina layer present. A stack such as this, with large surface area MoS_2_, would be an ideal electrolyte–electrode interfacial layer to promote ion insertion or photocathodes for water splitting, leading toward renewable solar-powered hydrogen fuel generation,^21^ or for highly sensitive gas sensing for pollution and environmental monitoring.^22^

## Future Work/Battery Devices

The heterostructures discussed here have potential to be used as battery electrodes with ion intercalation and deintercalation occurring between the MoS_2_ layers. Future work to demonstrate the performance of the heterostructure will include creating a pouch cell assembly, with the nanowire/2D heterostructures on the silicon substrate acting as a standalone electrode. Cyclic voltammetry and galvanostatic charge/discharge tests will be used to define the ionic mechanisms and specific capacity of the electrode which will be compared to other materials such as graphite. Beyond lithium ions, aluminum and sodium chemistry will be used to explore more environmentally and readily available ion battery technologies. Further investigations using flexible textured substrates will also be performed, for the application of flexible 2D batteries, as 2D materials are inherently flexible in their very nature.

## Conclusions

2D MoS_2_/SiNW heterostructures were fabricated, with MoS_2_ layer numbers tuned through control of the ALD cycle number, using a large-area, commercially scalable, two-step ALD/H_2_S anneal process. ALD alumina was used as an interfacial layer between MoS_2_ and nanowires. SEM verified that this two-step process results in no degradation of the silicon nanowires, whereby the MoO_3_ layer grown first protects the silicon from the H_2_S gas during anneal, while also allowing for the MoO_3_ to be converted to MoS_2_. The 100 ALD cycle samples demonstrate near bulk behavior by Raman with a peak separation of ∼25 cm^–1^ and show the lowest intensity in photoluminescence. The 15 ALD cycle samples demonstrated one to four MoS_2_ layers, with the presence of alumina reducing the Raman peak separation to 20 cm^–1^, indicating near monolayer growth. This was confirmed with TEM, where parallel growth is shown conformally across the silicon nanowire surface with two layers present. Photoluminescence demonstrated that the quality of the 15 ALD cycle samples is significantly improved in terms of defects, with a reduction in B/A intensity from 3 to 0.3. This work demonstrates a commercially compatible MoS_2_/SiNW heterostructure technique that is highly controllable and adaptable, allowing for substrate choice with or without an insulator, along with number of layers to be selected, lending the systems unique properties to applications in the energy storage and generation space.

## Data Availability

The data that support the findings of this study are available in 10.5258/SOTON/D2829
